# Central Serous Chorioretinopathy in a Case of Regressed Familial Retinoblastoma

**DOI:** 10.18502/jovr.v15i4.7802

**Published:** 2020-10-25

**Authors:** Saeed Karimi, Amir Arabi, Toktam Shahraki, Sare Safi

**Affiliations:** ^1^Ophthalmic Research Center, Research Institute for Ophthalmology and Vision Science, Shahid Beheshti University of Medical Sciences, Tehran, Iran; ^2^Department of Ophthalmology, Torfeh Medical Center, Shahid Beheshti University of Medical Sciences, Tehran, Iran; ^3^Ophthalmic Epidemiology Research Center, Research Institute for Ophthalmology and Vision Science, Shahid Beheshti University of Medical Sciences, Tehran, Iran

**Keywords:** Central Serous Chorioretinopathy, Recurrence, Retinoblastoma

## Abstract

**Purpose:**

To present a case of central serous chorioretinopathy (CSC) in association with regressed familial retinoblastoma.

**Case Report:**

A 23-year-old man with regressed unilateral familial retinoblastoma in his left eye presented with decreased vision of the left eye since two months ago. The patient had undergone chemotherapy and cryotherapy for the treatment of retinoblastoma 20 years ago. In the left eye, funduscopy disclosed regressed mass of retinoblastoma, inferonasal to the optic disc, and focal subfoveal neurosensory detachment. Optical coherence tomography (OCT) and fluorescein angiography revealed CSC. As there was no sign of recurrence of retinoblastoma and retinal findings did not show late-onset chemotherapy-related retinopathy, the patient was diagnosed with CSC and followed up. After two months, visual acuity of the left eye improved, and repeated macular OCT revealed absorption of the subretinal fluid.

**Conclusion:**

Subretinal fluid accumulation in a patient with regressed retinoblastoma is not always a sign of tumor recurrence or a treatment-related retinopathy.

##  INTRODUCTION

Central serous chorioretinopathy (CSC) is a retinal disease in which there is serous detachment of the neurosensory retina, typically restricted to the macular area. The disease is believed to be a disorder with multiple etiologies that lead to a common pathway of choroidal vascular abnormality.^[1]^ The pathogenesis is poorly known, and retrospective studies have recognized a number of risk factors. These include male sex,^[2]^ psychological tension,^[3]^ personality type A,^[4]^ corticosteroid use,^[5,6]^ gestation,^[7]^ and, infrequently, endocrine disorders (such as Cushing's syndrome)^[8]^ or tumors with steroid products.^[9]^

**Figure 1:**
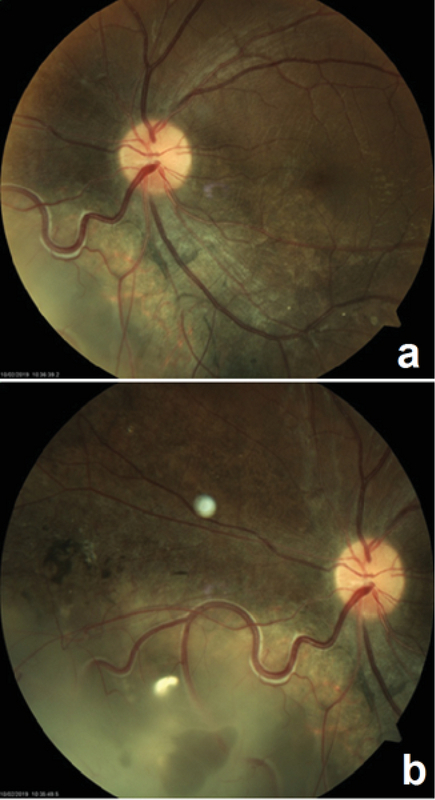
(a) Posterior fundus view of the left eye demonstrating the distance between the mass and fovea. (b) Regressed retinoblastoma with intralesional cavitation, inferonasal to the optic disc.

**Figure 2:**
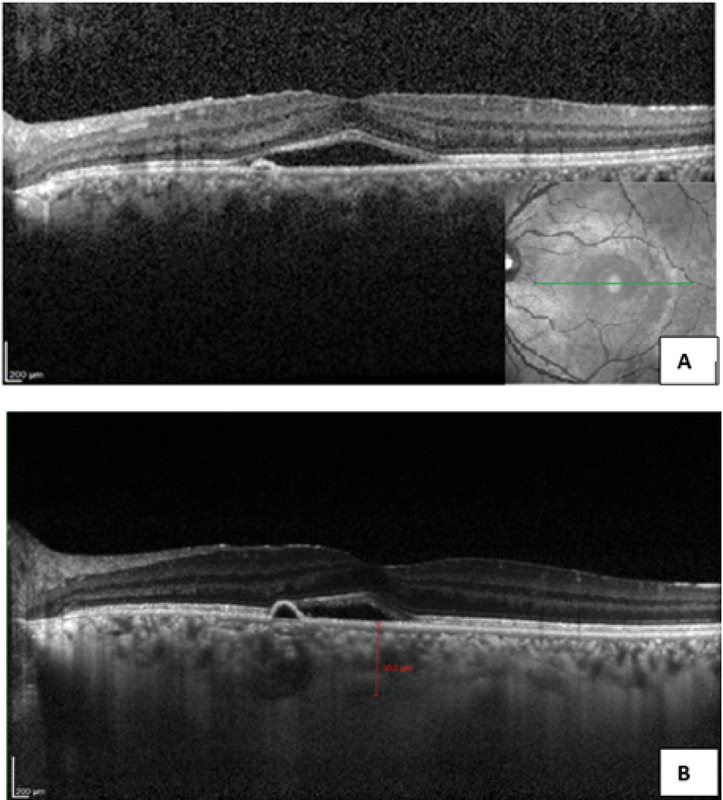
(A) SD-OCT of the left eye; neurosensory detachment with echo-free subretinal fluid adjacent to a well-defined PED. (B) EDI-OCT of the same eye; note the increased thickness of the choroid and congested vasculature.

**Figure 3:**
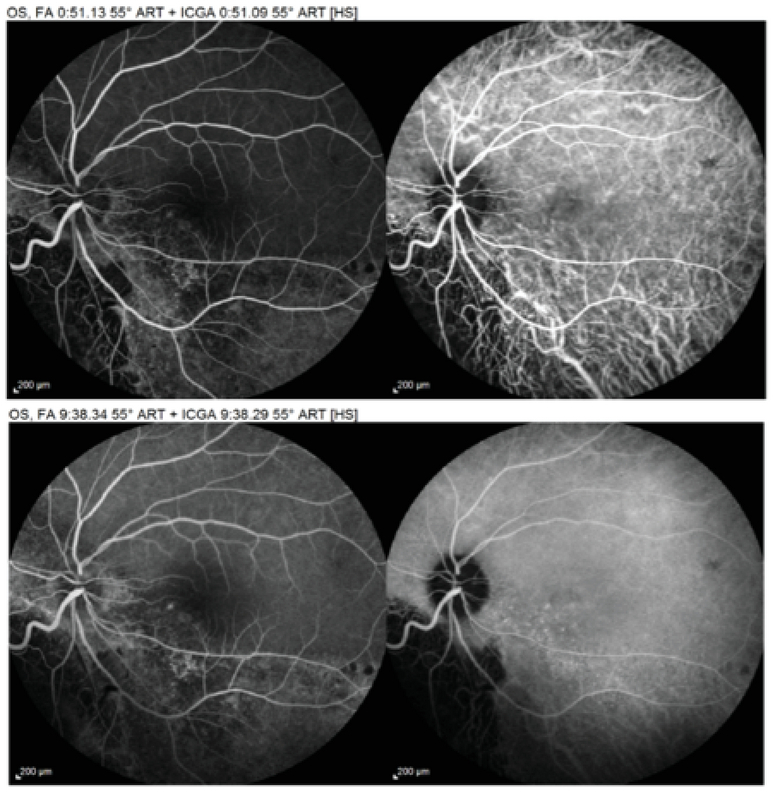
Early and late phases of fluorescein angiography (left) and ICG angiography (right) showing juxtafoveal subretinal leakage in addition to congestion of choroidal vessels.

**Figure 4:**
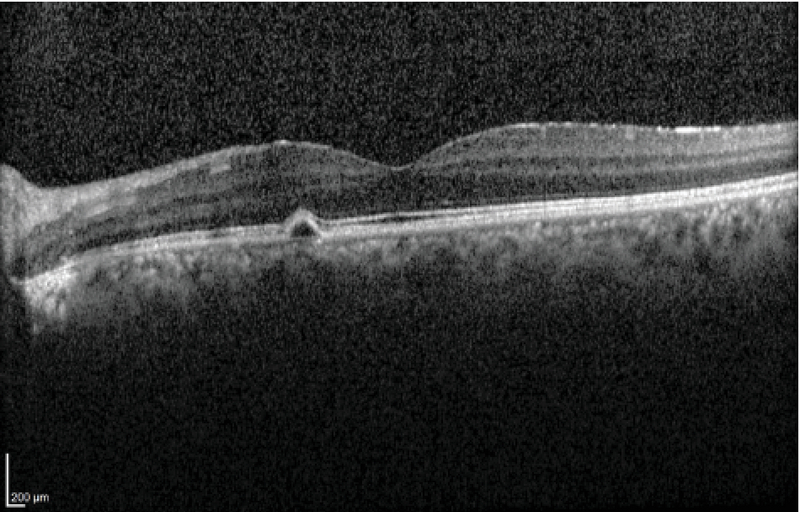
Macular SD-OCT of the same eye two months later shows resolved subretinal fluid.

Retinoblastoma is an inherited pediatric malignant neoplasia, assumed to be the most frequent intraocular malignancy in children.^[10]^ Concerns about patients with a history of retinoblastoma, especially those treated with external beam radiotherapy, are related to the risk of recurrence, secondary malignancies, and development of radiation retinopathy or retinal damage secondary to chemotherapy. All these conditions can develop even after a long period from the initial treatment.^[11,12,13]^

Here, we report a case of regressed retinoblastoma and coincidental CSC in the same eye. The association between regressed retinoblastoma and CSC has not been reported in the literature.

##  CASE REPORT

A 23-year-old man presented to the ophthalmology clinic with a two-month history of blurred vision in the left eye. The patient had a history of unilateral familial retinoblastoma in the left eye, which had been treated with cryotherapy and systemic chemotherapy 20 years before. Medical and drug histories were otherwise unremarkable. The patient had a positive family history of retinoblastoma, as his father and two siblings were diagnosed with bilateral familial retinoblastoma.

Ophthalmic examination showed a corrected distance visual acuity of 10/10 in the right eye and 5/10 in the left eye. Intraocular pressure was normal, and relative afferent pupillary defect was negative. Anterior segment examination showed normal findings. Fundus examination of the left eye revealed a white elevated regressed retinoblastoma mass with intralesional cavitation and focal subfoveal neurosensory detachment (Figure 1). The right eye was completely normal. Spectral-domain optical coherence tomography (OCT) revealed echo-free subfoveal fluid accumulation and a small pigment epithelial detachment. Additionally, enhanced depth imaging OCT of both eyes showed thick choroid and dilated choroidal vessels (Figure 2). Fluorescein angiography (FAG) of the left eye revealed an expansile leaking dot near the fovea in the mid-to-late phase. Indocyanine green angiography showed hyperpermeability of dilated choroidal vasculature (Figure 3). Based on clinical examination and paraclinical findings, a diagnosis of CSC coincidental with regressed unilateral familial retinoblastoma was made. As there was no sign of retinoblastoma recurrence or secondary malignancy and the clinical findings did not reflect late-onset retinopathy related to prior treatment, a close follow-up was planned. During the follow-up period, oral propranolol was prescribed. At the two-month re-examination, the best corrected visual acuity of the affected eye improved to 7/10, the retinoblastoma mass was unchanged, and the new macular OCT revealed absorption of the subretinal fluid (Figure 4).

##  DISCUSSION

Blurring of vision and new accumulation of subretinal fluid in a patient with history of retinoblastoma is usually suggestive of recurrent retinoblastoma, adverse effect of chemotherapy on the chorioretinal tissue, or late-onset radiation retinopathy. As there was no history of radiotherapy and intra-arterial chemotherapy in this case, ocular complications of these therapeutic interventions were not considered for the patient.

Posterior segment side effects of systemic chemotherapy have been studied for some drugs. Secondary retinopathy with visual loss develops with cisplatin use;^[14]^ however, retinal findings of cisplatin-induced retinopathy are different from those seen in our case. Moreover, retinal ischemia and subsequent neovascularization have been reported in a patient receiving chemotherapy with bleomycin, etoposide, and cisplatin.^[15]^ However, FAG of this patient revealed no sign of retinal ischemia or neovascularization. Subretinal fluid and choroidal changes have not been reported in ocular conditions associated with common agents used for systemic chemotherapy. The impact of intravenous chemotherapy on choroidal microvasculature was analyzed in a previous study, where the authors concluded that systemic chemotherapy cannot cause a significant change in choroidal thickness.^[16]^ These findings support the impression of increased choroidal thickness in the current case as a primary event.

Recurrence of retinoblastoma may be manifested by a new retinal tumor, vitreous seeding, subretinal seeding, or extraocular findings.^[17]^ Following systemic chemoreduction, the recurrence rate of retinal tumors ranges from 24 to 44%.^[18]^ The majority of new tumors will be detected within three years of initial retinoblastoma diagnosis. In a previous study, most retinal tumors recurred within five months of initiating chemoreduction, when the therapeutic period of chemotherapy had not ended.^[19]^ Although recurrence of retinoblastoma after 20 years is rare, complete retinal examination for any active retinal tumor was performed in our patient, and no sign of retinal recurrence was detected.

In eyes presenting with exophytic tumors with subretinal seeding at presentation, recurrence of subretinal seeding is a particular concern.^[17]^ Shields et al suggested an average interval of two months for the recurrence of subretinal seeding after completion of chemotherapy. However, calculated recurrence rates of 53 to 62% at one and three years, respectively, suggested that subretinal recurrence may happen after several years.^[18]^ The subretinal seeds may be free running or fixed to the external retinal surface.^[20]^ Retinal detachment, in addition to position-dependent ophthalmoscopic contours, is a characteristic finding in subretinal seeding of retinoblastoma.^[20]^ Features of subretinal fluid and its absorption on follow-up examinations ruled out the probability of recurrent subretinal seeding as the cause in our patient.

CSC is an ocular condition characterized by neurosensory detachment associated with pathological dilation of choroidal vessels. A number of systemic disorders, including autoimmune diseases, hypertension, allergic respiratory diseases,^[2,21]^ *Helicobacter pylori* infection, obstructive sleep apnea,^[22]^ and corticosteroid-releasing tumors have been reported to be related to the pathophysiology of CSC. Among these, no ocular malignancy has been shown to be associated with CSC. Although some conditions with choroidal origin such as radiation choroidopathy and choroidal neovascularization have been reported after treatment of retinoblastoma,^[23]^ in case of subretinal exudation in an eye with regressed retinoblastoma, retinoblastoma recurrence or secondary malignant tumors are considered prior to choroidal conditions.

This is the first report on the coincidence of CSC and retinoblastoma in the same eye. Although blurring of vision and accumulation of subretinal fluid in a case of retinoblastoma should be considered as indicators of recurrent retinoblastoma or treatment adverse effects, coincidence of CSC and retinoblastoma could be detected in our patient.
